# Dynamic profiles and subpopulation characteristics of extracellular vesicles in patients undergoing transcatheter pulmonary valve replacement

**DOI:** 10.20517/evcna.2026.24

**Published:** 2026-08-18

**Authors:** Runzhang Liang, Jiaxiong Wu, Naijimuding Abudurexiti, Jing Ling, Zirui Peng, Canxin Wang, Jinxin Li, Yong Zhang, Haiyun Yuan, Shusheng Wen

**Affiliations:** ^1^Department of Cardiovascular Surgery, Guangdong Provincial People’s Hospital (Guangdong Academy of Medical Sciences), Southern Medical University, Guangzhou 510080, Guangdong, China.; ^2^Guangdong Provincial Key Laboratory of South China Structural Heart Disease, Guangzhou 510080, Guangdong, China.; ^3^Department of Cardiovascular Surgery, Guangdong Provincial Hospital of Chinese Medicine, The Second Affiliated Hospital of Guangzhou University of Chinese Medicine, Guangzhou 510120, Guangdong, China.; ^4^Department of Cardiovascular Surgery, Guangdong Cardiovascular Institute, Guangdong Provincial People’s Hospital, Guangdong Academy of Medical Sciences, Guangzhou 510080, Guangdong, China.; ^#^These authors contributed equally to this work.

**Keywords:** Proximity barcoding assay, extracellular vesicle, protein, transcatheter pulmonary valve replacement

## Abstract

**Aim:** Transcatheter pulmonary valve replacement (TPVR) is a crucial intervention for severe pulmonary regurgitation (PR). Extracellular vesicle (EV) surface proteins reflect cardiovascular pathology. This study employed a proximity barcoding assay (PBA) to dynamically monitor EV protein profiles during TPVR.

**Methods:** In this study, 40 biological plasma samples were analyzed in triplicate, including 10 from healthy controls (*N* group) and 30 from TPVR patients across three time points: preoperative (T1 group), 1 day postoperative (T2 group), and 1 week postoperative (T3 group). We performed combinatorial and functional analyses of membrane proteins across different groups and evaluated their predictive performance. EV subpopulations were clustered and characterized.

**Results:** Compared with the *N* group, patients in the T1 group exhibited significantly downregulated expression of DSCAML1, ALCAM, and LAG3 proteins, while CD8A, NECTIN1, and LGR5 were significantly upregulated (*P* < 0.001; AUC: 0.94-1.00). Postoperatively, the expression of ALCAM and LAG3 rapidly rebounded. The ALCAM&CD36&DSCAML1&PECAM1 combination was significantly elevated in the control group, whereas CD36&CD8A&DSCAML1&TACSTD2 was reduced. Gene Ontology (GO) analysis indicated associations with response to stress, side of membrane, and protein binding pathways. Kyoto Encyclopedia of Genes and Genomes (KEGG) analysis revealed enrichment in the phosphatidylinositol 3-kinase (PI3K)/protein kinase B (PKB/AKT) signaling pathway. Subpopulation analysis revealed that Cluster 1 was significantly elevated, whereas Cluster 2 was markedly decreased in the disease group. Additionally, Cluster 4 dominated in the T3 group. Furthermore, CD8A is most abundant in Cluster 1, while DSCAML1 dominates in Clusters 2 and 4.

**Conclusion:** This study provides the first dynamic profiles and subpopulation characteristics of plasma EVs in patients undergoing TPVR, establishing a molecular atlas of perioperative right ventricular remodeling

## INTRODUCTION

Congenital heart disease (CHD) is the most common congenital malformation, with an incidence of approximately 0.29%^[1]^. 20% of CHD patients develop right ventricular outflow tract obstruction (RVOTO), including tetralogy of Fallot (TOF), pulmonary atresia with ventricular septal defect (PA/VSD), pulmonary stenosis (PS), double outlet right ventricle (DORV), and persistent truncus arteriosus (PTA)^[2]^. Surgical correction for these conditions requires right ventricular outflow tract (RVOT) reconstruction to relieve the obstruction. Following surgical treatment, 48% of patients develop pulmonary regurgitation (PR) immediately, and 85% develop PR within 2 years^[3]^. Long-term PR leads to increased right ventricular (RV) volume overload, which in turn induces myocardial fibrosis and hypertrophy, causing RV remodeling and eventually resulting in right heart failure (RHF), atrial or ventricular arrhythmias, and even sudden cardiac death, thereby necessitating pulmonary valve replacement (PVR)^[4,5]^. Currently, PVR encompasses both surgical pulmonary valve replacement (SPVR) and transcatheter pulmonary valve replacement (TPVR). These procedures aim to correct severe PR, thereby alleviating patient symptoms, reversing RV remodeling, improving cardiac function, and ultimately extending patient survival^[6]^. Studies have shown that TPVR is associated with a low incidence of perioperative complications and a shorter hospital stay^[7]^. Given the substantial inter-individual variability in RV remodeling after TPVR, profiling the perioperative molecular landscape of plasma exosomes could facilitate the discovery of key molecules that regulate RV reverse remodeling and deepen our understanding of its pathophysiological mechanisms. Extracellular vesicles (EVs), as key carriers of intercellular communication, have surface membrane protein profiles that can dynamically reflect cardiovascular pathological conditions^[8]^. These nanovesicles, ranging in diameter from 30 to 150 nm, carry proteins, nucleic acids, and lipids. By leveraging their surface proteins for targeted interactions, they can deliver signaling molecules to specific tissues or organs, thereby playing a pivotal role in the regulation of various physiological and pathological processes. Studies have shown that elevated expression of CD31+/Annexin V+ microparticles is associated with an increased risk of mortality in patients with CHD and can serve as an independent predictor of prognosis^[9,10]^. Elevated levels of EV contents such as CCL2, CCL7, and IL-6 indicate an enhanced cellular inflammatory response^[11]^. Consequently, exosomes provide a valuable window into the molecular landscape and pathophysiological mechanisms of cardiovascular diseases (CVD)^[12]^.

However, conventional bulk-level analyses lack the resolution to dissect EV subpopulation heterogeneity, rendering them incapable of detecting subtle dynamic changes associated with RV remodeling. The Proximity Barcoding Assay (PBA) enables the study of the composition and abundance of exosomal membrane proteins at the single-EV level, facilitating the dissection of differences among EVs secreted by cells of various tissue origins and distinct physiological states^[13]^. Furthermore, PBA provides a powerful tool for dissecting the molecular landscape of EV surface proteins, which can help uncover disease pathogenesis^[13]^.

This study is the first to apply PBA technology for dynamic monitoring of plasma EVs in TPVR patients during the perioperative period. By elucidating the surface protein profiles and subpopulation characteristics of plasma EVs, this study aims to provide a molecular atlas of RV remodeling.

## METHODS

### Study subjects

This study enrolled 10 patients with postoperative PR secondary to CHD who underwent TPVR via a trans-thoracic approach at Guangdong Provincial People’s Hospital, constituting the experimental group. Inclusion criteria were as follows, (1) moderate to severe PR confirmed by echocardiography; (2) evidence of RVOT dysfunction, meeting at least one of the following, right ventricular end-diastolic volume indexed (RVEDVI) ≥ 130 mL/m^2^, right ventricular ejection fraction (RVEF) < 45%, QRS width ≥ 160 ms, sustained atrial or ventricular arrhythmias, or moderate or greater tricuspid regurgitation; (3) mean pulmonary artery pressure ≤ 30 mmHg; and (4) main pulmonary artery length ≥ 20 mm and diameter ≥ 16 mm. Exclusion criteria were as follows, (1) pulmonary hypertension or anatomical contraindications (e.g., abnormal RVOT position, coronary artery anomalies); (2) acute heart failure; (3) active infective endocarditis or a history of prior infective endocarditis; (4) allergy to aspirin, heparin, nickel-titanium alloy, or contrast agents; (5) active rheumatic disease; (6) infectious diseases such as hepatitis B, hepatitis C, HIV, or syphilis. Additionally, 10 healthy individuals were selected as a control group. The study was conducted in accordance with the Declaration of Helsinki and approved by the Ethics Committee of the Guangdong Provincial People’s Hospital (approval number: QX2021-011-02). All participants provided written informed consent prior to enrollment.

### Sample collection

Venous blood (12 mL) was collected from each of the 10 patients undergoing TPVR at three time points: preoperatively (T1 group), 1 day postoperatively (T2 group), and 7 days postoperatively (T3 group). For the control group (*N* group), venous blood (12 mL) was collected simultaneously from 10 healthy individuals. Each plasma sample was technically replicated in triplicate for PBA analysis, yielding a total of 120 technical measurements. All samples were drawn into ethylenediaminetetraacetic acid (EDTA) anticoagulant tubes and processed promptly, or temporarily stored at 4 °C and processed within 4 h. The samples were centrifuged at 1,500 × *g* for 20 min at 4 °C to remove cellular component (CC). The plasma supernatant was carefully transferred to a new 1.5 mL centrifuge tube. Subsequently, the supernatant was centrifuged at 3,000 × *g* for 15 min at 4 °C to thoroughly remove residual blood cells. The plasma supernatant was then carefully aspirated, aliquoted, and stored at -80 °C.

### EV isolation

For EV identification by Nanoparticle Tracking Analysis (NTA), Transmission Electron Microscopy (TEM), and Western Blot (WB), separate plasma aliquots from three healthy controls were pooled and processed by ultracentrifugation independently of the samples used for PBA analysis. NTA, TEM, and WB were performed for EV identification. Frozen plasma was thawed completely at 4 °C and kept on ice. Samples were centrifuged at 500 × *g* for 10 min at 4 °C, followed by centrifugation at 20,000 × *g* for 20 min at 4 °C. The supernatant was then centrifuged at 100,000 × *g* for 70 min at 4 °C. The resulting pellet was resuspended in 40 mL pre-chilled phosphate-buffered saline (PBS) and centrifuged again at 100,000 × *g* for 70 min at 4 °C.

For PBA analysis, EVs were prepared by size-exclusion chromatography and ultrafiltration (UF) as follows. (1) Plasma Pretreatment: Thaw the plasma and filter it through a 0.8 μm filter to remove large particles. Rinse the filter with 250 μL of PBS, combine the filtrate, and adjust the volume to 1 mL for subsequent steps; (2) Column Equilibration: Retrieve the gel exclusion column (Exosupur® ES9P14e/ES9P11e, Echo Biotech, China) stored at 4 °C, and wash it with 10 mL of 0.1 M PBS to equilibrate the column environment; (3) Sample Loading: After the liquid in the column has completely drained, load the 1 mL pretreated plasma onto the column, allowing it to pass through the resin slowly; (4) Elution & Collection: Once the sample has fully entered the resin, elute with 0.1 M PBS, collecting the eluate in two fractions of 1 mL each (total 2 mL). During this phase, EVs elute with the PBS solution; (5) UF & Lysis: Combine the eluates in a 100 kDa molecular weight cut-off Amicon® Ultra spin filter (Merck, Germany), centrifuge at 4,000 × *g* for 2 min to concentrate to approximately 200 μL. Add 700 μL of lysis buffer, mix thoroughly, and incubate for 2 min to obtain the lysate for PBA analysis.

### NTA

EV samples were diluted with PBS to a detection concentration of 1 × 10^8^/mL for analysis. First, the instrument flow rate was calibrated using fluorescent silica microspheres with a known concentration and a diameter of 250 nm by correlating detection time with particle count. Under the same pressure conditions, the actual concentration of the EV sample was calculated based on this flow rate and particle count. Meanwhile, standard silica microspheres with diameters of 68, 91, 113, and 155 nm were used to establish a standard curve of scattered light intensity versus particle size. The scattered signals from the EV samples were converted to size data using this curve, thereby obtaining their final size distribution.

### TEM

10 µL of EV suspension was deposited onto a copper mesh and incubated at room temperature for 10 min to facilitate adsorption. The mesh was then rinsed with sterile distilled water, and residual liquid was removed with absorbent paper. Negative staining was performed by applying 10 µL of 2% uranyl acetate for 1 min; excess stain was removed, and the grid was dried for 2 min under an incandescent lamp. The prepared grid was then examined under a transmission electron microscope (H-7650, Hitachi Ltd., Tokyo, Japan) at an accelerating voltage of 80 kV. This protocol enhances contrast through negative staining and reduces sample damage via low-voltage imaging, thereby clearly revealing the ultrastructural details of the EVs.

### WB analysis

The protein concentration of EVs was determined using the Bicinchoninic Acid assay. Based on the calculated concentration, a loading amount of 10-30 µg was mixed with 5× SDS loading buffer, vortexed, and denatured by heating at 95 °C for 5 min. Denatured samples were then subjected to SDS-PAGE electrophoresis. Target gel regions containing exosomal markers Alix, CD9, CD81, and the negative control Calnexin were excised and transferred onto PVDF membranes via electroblotting. After blocking the membranes with 3% BSA, they were sequentially incubated with primary and secondary antibodies. Finally, the target proteins were visualized and analyzed through development, fixation, and exposure.

### PBA processing and high-throughput sequencing

The surface proteins on EV were profiled using the PBA (ExoSeek® panel260, Secretech, Shenzhen, China)^[13]^. The PBA procedure was described in the PBA method paper^[13]^. Briefly, we follow the steps below. The PBA detection leverages a panel of 260 oligonucleotide-labeled antibodies to detect EV proteins simultaneously. Each antibody was conjugated with oligonucleotides consisting of a unique protein tag to mark protein type and a molecular tag to label duplicate detection. The EVs were captured on a surface with cholera toxin subunit B (CTB) coating. The antibody-labeled oligonucleotides located on the same EV were assigned the same unique EV tag. The DNA sequences comprising barcoding information of EV tag - protein tag - molecule tag were prepared into a sequencing library for each sample. The libraries were prepared for PE150 sequencing with the DNBSEQ-T7 platform (MGI, Shenzhen, China) or NovaSeq S4 (Illumina, USA). The sequencing raw data in bcl files were transferred into fastq files with DNA sequences using the bioinformatic tools MegaBolt for the MGI instrument or bcl2fastq for the Illumina platform.

### EV surface protein detection

This approach utilizes PBA technology to achieve combinatorial detection of membrane protein assemblies on individual EVs. It facilitates the exploration of co-expression and co-localization patterns among multiple membrane proteins at the single-EV level, thereby elucidating their potential collaborative involvement in intricate biological processes. In this study, we enumerated the number of vesicles containing each possible protein combination within each sample. Based on the single EV protein expression matrix for each sample, we initially categorized EVs according to the number of protein types they carried into four groups. EVs carrying only one protein were designated as combination_1; those carrying two proteins as combination_2; those carrying three proteins as combination_3; and EVs carrying four or more proteins as combination_4More. For each EV category, we profiled the expressed protein combinations and their respective abundances. Subsequently, we performed count per million (CPM) normalization on the EV protein combination data for each category. CPM normalization is calculated by dividing the protein expression level by the total expression level of a sample and then multiplying by 10^e6^.

### Flow cytometric analysis

Plasma EVs were isolated as described above. For surface protein staining, purified EVs were resuspended in PBS and incubated with the following fluorochrome-conjugated antibodies^[14]^, PE anti-human LGR5, APC/Cyanine7 anti-human CD8a, ABflo® 647 anti-human CD111 (targeting NECTIN1), PE/Cyanine7 anti-human CD223 (targeting LAG-3), and ABflo® 488 anti-human CD166 (targeting ALCAM). All antibodies were used at the dilutions recommended by the manufacturer. The staining reaction was carried out at 4 °C for 30 min in the dark. After incubation, the stained EV samples were diluted with PBS to a final volume of 200 µL and analyzed immediately on a flow cytometer (FACSCanto^TM^ II, BD Biosciences). To discriminate EV events from background noise, a threshold was established based on the side scatter (SSC) and forward scatter (FSC) properties of size-calibrated reference beads (100 and 200 nm). At least 10,000 EV events were acquired for each sample. Data analysis was performed using FlowJo software (version 10). The percentage of EVs positive for each target protein was determined by gating against the corresponding isotype controls and fluorescence-minus-one (FMO) controls.

### EV subpopulation analysis

We conducted subpopulation and subpopulation marker identification for EVs using large-scale vesicle datasets. The analysis of EV subpopulations was carried out in the following steps: (1) Data Cleaning: We performed rigorous cleaning on massive vesicle datasets, filtering out low-abundance vesicles. The criterion retained vesicles that contained at least two distinct proteins, each with a count ≥ 2. To reduce computational burden, we randomly sampled 1/9 of vesicles from each sample for subpopulation identification. Proportional downsampling was applied uniformly across all samples to reduce computational bias caused by uneven EV counts while preserving relative subpopulation structure; (2) Data Normalization: We mitigated excessive dispersion in EV data to achieve a more uniform vesicle distribution. For the EV expression vector, the specific vesicle normalization formula is as follows: ev (normalization) = log_10_ (*cpm* + 1); (3) Scale: We applied scaling to the EV data features (i.e., protein vectors) to eliminate the influence caused by unit and scale differences between features. The method employed was the z-scale method; (4) Feature Extraction: We utilized Principal Component Analysis (PCA) to further reduce data dimensionality, obtaining the principal dimensions that contribute to variation and diminishing the impact of dimensions with minor contributions; (5) EV Subpopulation Identification: We employed the self-organizing map (SOM) algorithm provided by FlowSOM^[15]^ to cluster single EV data, identifying EV subpopulations. Clustering robustness was evaluated through repeated subsampling and independent FlowSOM runs to ensure consistent identification of major EV subpopulations. This includes calculating the proportion of each subpopulation and comparing protein expression levels across subpopulations; (6) EV Subpopulation Visualization: We utilized t-distributed stochastic neighbor embedding (t-SNE) and Uniform Manifold Approximation and Projection (UMAP) dimensionality reduction and visualization algorithms to graphically represent single EV protein expression data.

### EV proteome data analysis and statistical analysis

The sequencing reads were analyzed with the software EVisualizer® decoding package (version 1.0, Secretech, Shenzhen, China) to generate EV ID-protein expression datasets as PBA raw data and then to analyze protein expression, combinations, and EV subpopulations. Briefly, the algorithm used in EVisualizer® is as follows. Based on the raw data, the total number of detected EVs and proteins was summarized as mean ± standard error (SE). The total expression of each protein was summarized as a protein expression dataset, which was subsequently normalized via the trimmed mean of M-values (TMM) algorithm to obtain a normalized total protein expression dataset. The co-expression of two or more proteins on the same single EVs was defined as a protein combination dataset. CPM normalization was performed for the protein combination dataset. For analysis of differentially expressed proteins (DEPs) and differentially expressed protein combinations (DEPC), we performed statistical analysis as follows. We examine the normality of data with the Shapiro-Wilk test. We examine the homogeneity of data with the *F*-test or Bartlett’s test. When comparing data of two groups, we use Student’s *t*-test if the data show normal distribution and homogeneous variance. The Mann-Whitney *U* test is used if the data is not normally distributed. The Welch’s *t*-test is used for data with normal distribution but not homogeneous variance. When comparing more than two groups (> 2 variables or categories), we choose the Analysis of Variance (ANOVA) test for a dataset with a normal distribution and homogeneous variance. The significant differential expression is analyzed with Duncan’s test. If the input data doesn’t have a normal distribution or homogeneous variance, the non-parametric Kruskal-Wallis test was used instead. The significant differential expression is analyzed with the Pairwise Wilcoxon Rank Sum Test. We leveraged the Benjamini-Hochberg (BH) method to adjust the *P*-values. Adjusted *P*-values (false discovery rate, FDR) < 0.05 were considered statistically significant. We also constructed the receiver operating characteristic (ROC) curves and calculated the area under the curve (AUC) values using the pROC package (v. 1.18.0)^[16]^. Spearman correlation analysis was used to assess the associations between EV protein levels and clinical parameters in TPVR patients. To generate EV subpopulations, the unsupervised FlowSOM algorithm was used^[15]^. The t-SNE and UMAP methods could be employed for plotting EV subpopulations^[17,18]^. All analyses were conducted using R software and the EVisualizer® platform (version 1.0, Secretech, Shenzhen, China).

## RESULTS

### Identification of EV

EV identification was conducted using newly processed healthy-control plasma samples that were independent of the PBA samples. NTA, TEM, WB were performed for EV identification. NTA showed a predominant particle population in the small-EV size range, with a modal diameter of 84.4 nm and a mean diameter of 131.7 ± 0.7 nm [Figure 1A]. The particle concentration was 4.33 × 10^11^ ± 5.30 × 10^9^ particles/mL [Figure 1A]. TEM revealed membrane-bound vesicular structures with a round to cup-shaped morphology [Figure 1B]. WB detected the EV-associated proteins CD9, CD81, and ALIX in the EV preparation, whereas these markers were not detected in the plasma supernatant [Figure 1C]. In contrast, the endoplasmic reticulum marker Calnexin was not detected in the EV but showed weak expression in the plasma supernatant. NTA, TEM, WB were performed solely for EV identification.

**Figure 1:**
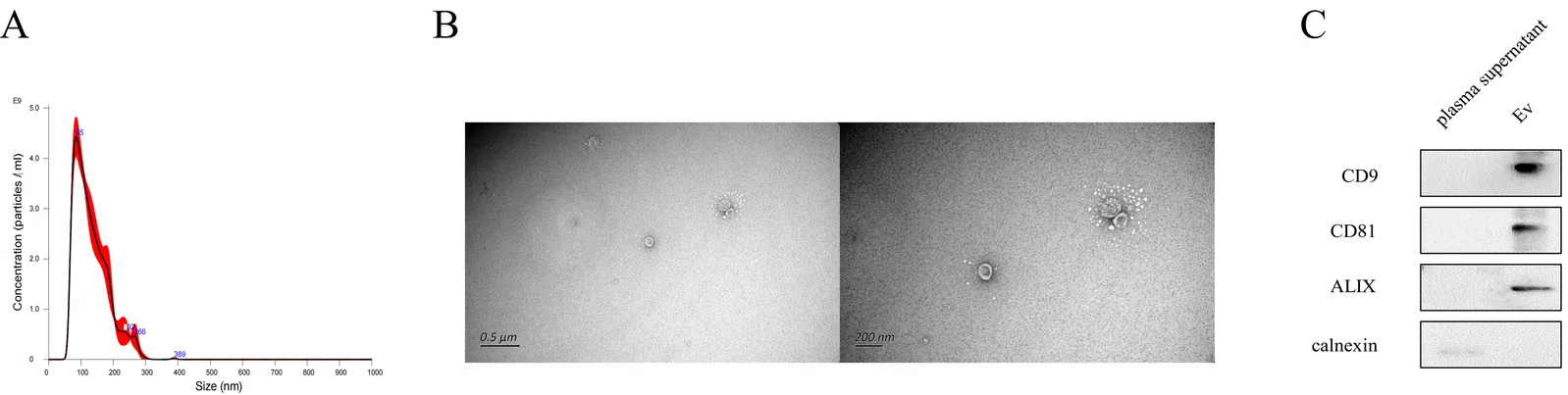
Identification of EV from the mixed sample of healthy controls, being independent of the PBA samples and without biological duplicates. (A) Particle size distribution analysis of EVs by NTA; (B) Representative TEM images showing membrane-bound vesicular structures with round to cup-shaped morphology. Scale bars, 0.5 μm and 200 nm; (C) Western blot analysis of EV-associated proteins CD9, CD81, ALIX, and alnexin. EV: Extracellular vesicle; NTA: nanoparticle tracking analysis; TEM: Transmission Electron Microscopy.

### EV surface protein detection

We performed a differential expression analysis of proteins between the *N* and T1 groups, and generated a heatmap to visualize the DEPs between EVs from the T1 group and those from the *N* group [Figure 2A]. Specifically, the proteins DSCAML1, ALCAM, and LAG3 were markedly downregulated in the T1 group (*P* < 0.001), while CD8A, NECTIN1, and LGR5 were significantly upregulated (*P* < 0.001) [Figure 2B-G]. Subsequent ROC curve validation showed AUC values of 0.94, 0.96, and 0.96 for DSCAML1, ALCAM, and LAG3, respectively [Figure 2H-J]. Conversely, the AUC values for CD8A, NECTIN1, and LGR5 were 0.96, 1.00, and 1.00, respectively [Figure 2K-M]. We then performed functional enrichment analysis on these DEPs. Biological process (BP) analysis indicated that these proteins are primarily involved in cellular processes [Figure 2N]. Molecular function (MF) analysis showed that these proteins are mainly associated with binding [Figure 2O]. CC analysis revealed that the proteins are predominantly located in integral components of the plasma membrane, intrinsic components of the membrane, and integral components of the membrane [Figure 2P]. Furthermore, Kyoto Encyclopedia of Genes and Genomes (KEGG) enrichment analysis demonstrated that these proteins are significantly enriched in pathways such as the intestinal immune network for IgA production, tight junction, and natural killer cell-mediated cytotoxicity [Figure 2Q].

**Figure 2:**
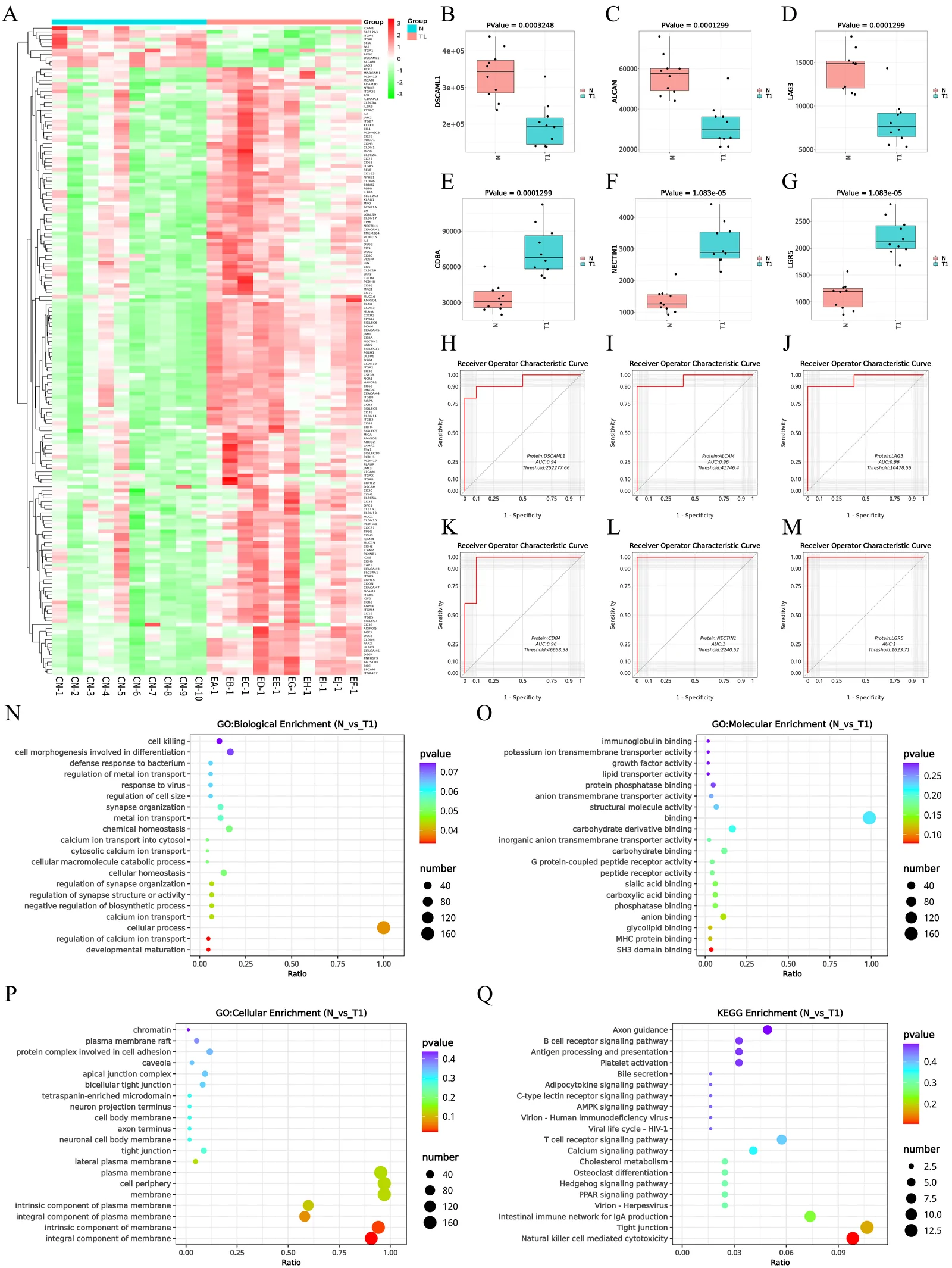
Differential expression analysis of individual proteins between the *N* and T1 groups. The heatmap displays the DEPs in EVs between the *N* and T1 groups (A). Histograms of DEPs (B-G). ROC curve analyses of DEPs (H-M). GO pathway enrichment analysis of DEPs (N-P). KEGG pathway enrichment analysis of DEPs (Q). DEPs: Differentially expressed proteins; EV: extracellular vesicle; GO: Gene Ontology; KEGG: Kyoto Encyclopedia of Genes and Genomes; ROC: Receiver operating characteristic.

We compared the protein expression profiles between the preoperative and postoperative groups, and generated a heatmap of DEPs [Supplementary Figure 1A]. The expression levels of ALCAM and LAG3 proteins on postoperative day 1 were significantly higher than preoperative levels (*P* < 0.05) [Supplementary Figure 1B and C]. Conversely, CTLA4 protein expression on postoperative day 1 was significantly lower than preoperative (*P* < 0.01) [Supplementary Figure 1D]. Subsequent ROC curve analysis demonstrated that the AUC values for ALCAM, LAG3, and CTLA4 proteins were 0.80, 0.79, and 0.87, respectively [Supplementary Figure 1E-G]. We further performed functional enrichment analysis on these DEPs. Gene Ontology (GO) analysis revealed that, in terms of BP, these proteins are primarily involved in immune system processes and the regulation of T cell differentiation; in MF, they predominantly participate in transmembrane signaling receptor activity, cytokine receptor activity, and immune receptor activity; and in CC, they are mainly associated with integral components of the plasma membrane, intrinsic components of membranes, and integral membrane components [Supplementary Figure 1H-J]. KEGG pathway enrichment analysis indicated that these proteins are significantly involved in the side of the membrane and the external side of the plasma membrane [Supplementary Figure 1K]. Additionally, comparative analysis was performed between the T1 and T3 groups [Supplementary Figure 2A-K].

Subsequently, multiprotein combination analysis revealed that combinations such as ALCAM&CD36&DSCAML1&PECAM1, ALCAM&CD8A&DSC1&DSCAML1, ALCAM&DSCAML1 &ITGA1&ITGB1, and ALCAM&CD36&DSCAML1&ITGA1 were expressed at significantly higher levels in the *N* group compared to the T1 group (*P* < 0.001) [Figure 3A-E], The expression levels of the combination of CD36&CD8A&DSCAML1&TACSTD2 and CD8A&DSCAML1&KIT&SIGLEC6 were significantly lower in the *N* group compared to the T1 group (*P* < 0.05) [Figure 3F and G]. ROC curve analysis showed that the AUC values for these combinations all exceeded 0.94 [Figure 3H-K]. BP analysis indicated that these proteins are primarily involved in defense response, response to stress, and immune system processes [Figure 3L]. MF analysis showed that these proteins mainly participate in protein binding and cytokine binding [Figure 3M]. CC analysis revealed that the proteins are predominantly located on the cell surface, on the side of the membrane, and on the external side of the plasma membrane [Figure 3N]. KEGG enrichment analysis demonstrated that these proteins are significantly enriched in pathways such as the PI3K-Akt signaling pathway, neutrophil extracellular trap formation, and hematopoietic cell lineage [Figure 3O].

**Figure 3:**
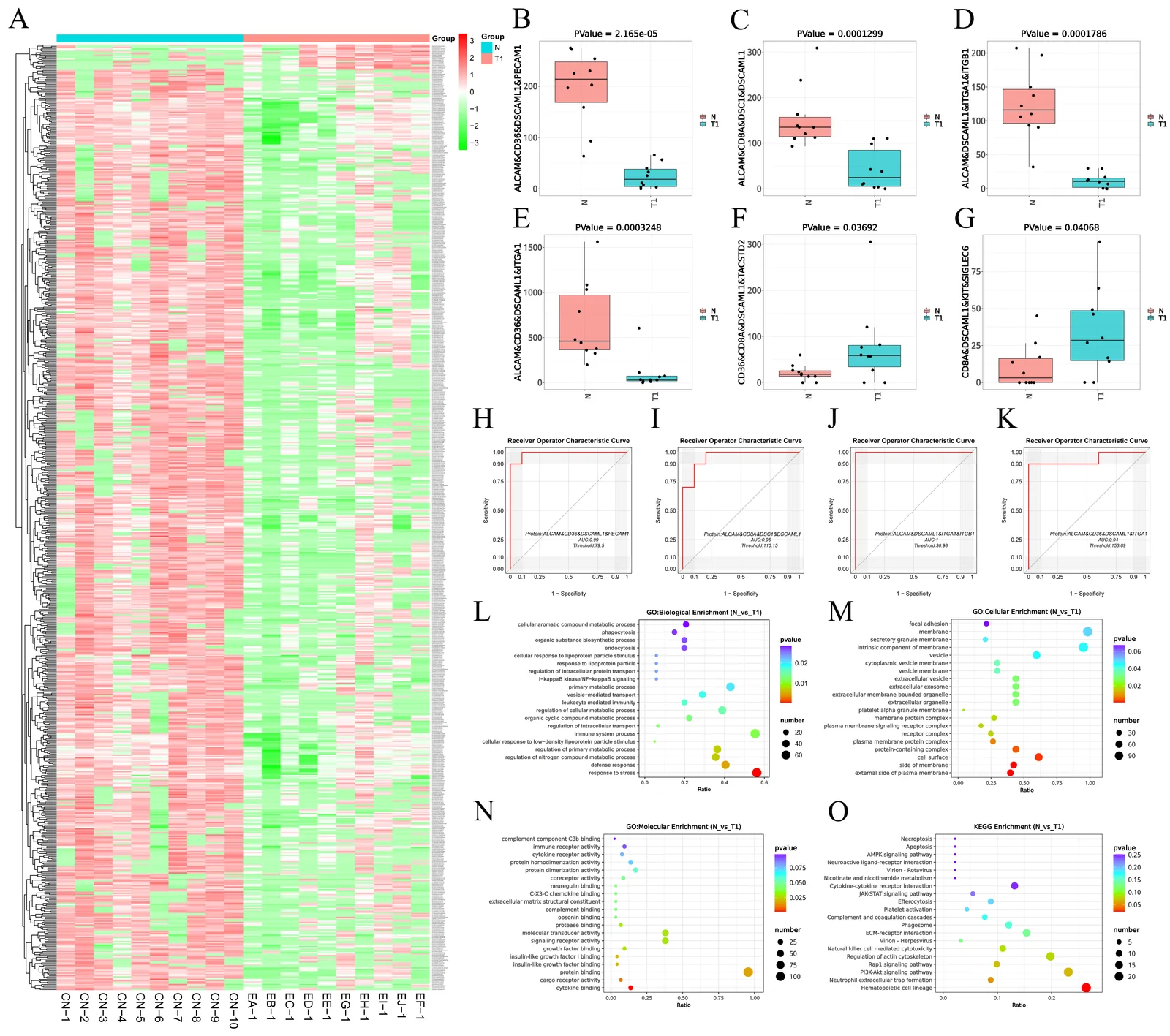
Differential expression analysis of protein combinations between the *N* group and the T1 group. The heatmap displays the differentially expressed protein combinations in EVs between the *N* and T1 groups (A). Histograms of differentially expressed protein combinations (B-G). ROC curve analyses of differentially expressed protein combinations (H-K). GO pathway enrichment analysis of differentially expressed protein combinations (L-N). KEGG pathway enrichment analysis of differentially expressed protein combinations (O). GO: Gene Ontology; KEGG: Kyoto Encyclopedia of Genes and Genomes; ROC: Receiver operating characteristic.

Furthermore, comparative analysis between the T1 and T2 groups revealed that combinations such as CD36&CD8A&DSCAML1&FCGR1A, CD36&CD8A&DSCAML1&Ly6G, ALCAM&CD36& CD8A&DSCAML1, and CD36&DSCAML1&ITGA1&ITGA2 were expressed at significantly higher levels in the postoperative group than in the preoperative group (*P* < 0.001) [Supplementary Figure 3A-E], and ROC curve analysis showed that the AUC values for these combinations all exceeded 0.85 [Supplementary Figure 3F-I]. BP analysis revealed that these proteins are primarily involved in complement activation, the alternative pathway, and the regulation of phagocytosis [Supplementary Figure 3J]. MF analysis indicated that these proteins are mainly associated with C-X3-C chemokine binding and protease binding [Supplementary Figure 3K]. CC analysis showed that the proteins are predominantly located in phagocytic vesicles, the cell surface, and intrinsic membrane components [Supplementary Figure 3L]. KEGG enrichment analysis demonstrated significant enrichment in pathways such as the Hematopoietic cell lineage and Efferocytosis [Supplementary Figure 3M]. Additionally, we performed a comparative analysis between the T1 and T3 groups [Supplementary Figure 4A-K]. Based on the co-expression data of proteins detected at the single EV level, we performed PCA and Multidimensional Scaling (MDS) on the co-expression patterns of various protein combinations, including single proteins [Supplementary Figure 5A and B], pairs of proteins [Supplementary Figure 5C and D], triples of proteins [Supplementary Figure 5E and F], and groups of four or more proteins [Supplementary Figure 5G and H].

#### Flow cytometry analysis

To validate the previous findings, we performed flow cytometric analysis of plasma EVs from healthy controls (*N*, *n* = 7), preoperative patients (T1, *n* = 5), and postoperative day 1 patients (T2, *n* = 7) for five proteins: ALCAM, LGR5, LAG3, NECTIN1, and CD8a. However, flow cytometric analysis revealed that none of the five surface proteins showed statistically significant differences among the *N*, T1, and T2 groups. Detailed data are presented in Supplementary Figures 6 and 7.

#### Correlations between EV proteins and clinical indices

Spearman correlation analysis was performed to evaluate the associations between differentially expressed EV proteins and preoperative as well as postoperative clinical parameters in TPVR patients [Table 1]. Preoperatively, LGR5 and NECTIN1 showed moderate positive correlations with RVEF (r = 0.668 and 0.649, both *P* < 0.05). left ventricular ejection fraction (LVEF) was positively correlated with DSCAML1, LAG3, and ALCAM (r = 0.695, 0.695, and 0.652, respectively; all *P* < 0.05). Left ventricular end-systolic volume (LVESV) also correlated positively with ALCAM, DSCAML1, and LAG3 (r = 0.733, 0.721, and 0.721; *P* < 0.05). QRS duration was positively associated with DSCAML1 and LAG3 (r = 0.713 for both; *P* < 0.05). In contrast, RV thickness, RV, and the RVEDV/LVEDV ratio were negatively correlated with NECTIN1 and/or LGR5 (r ranging from -0.646 to -0.732; all *P* < 0.05). Postoperatively, LVEF was negatively correlated with CD8A, NECTIN1, and LGR5 (r = -0.755, -0.706, and -0.693, respectively; all *P* < 0.05). QRS remained positively correlated with DSCAML1 and LAG3 (r = 0.669 for both; *P* < 0.05). Notably, RVSP showed strong negative correlations with CD8A, NECTIN1, and LGR5 (r = -0.833, -0.809, and -0.772, respectively; all *P* < 0.01).

**Table 1:** Correlations of EV proteins with preoperative and postoperative clinical indices

**Clinical indices**	**Proteins**	**Correlation coefficient**	***P* Value**
Preoperative			
RVEF	LGR5	0.668	0.035
RVEF	NECTIN1	0.649	0.042
LVEF	DSCAML1	0.695	0.026
LVEF	LAG3	0.695	0.026
LVEF	ALCAM	0.652	0.041
LVESV	ALCAM	0.733	0.016
LVESV	DSCAML1	0.721	0.019
LVESV	LAG3	0.721	0.019
QRS	DSCAML1	0.713	0.021
QRS	LAG3	0.713	0.021
RV_thickness	NECTIN1	-0.732	0.016
RV_thickness	LGR5	-0.646	0.043
RV	NECTIN1	-0.693	0.026
RV	LGR5	-0.669	0.035
RVEDV/LVEDV	LGR5	-0.673	0.033
Postoperative			
LVEF	CD8A	-0.755	0.012
LVEF	NECTIN1	-0.706	0.023
LVEF	LGR5	-0.693	0.026
QRS	DSCAML1	0.669	0.035
QRS	LAG3	0.669	0.035
RVSP	CD8A	-0.833	0.003
RVSP	NECTIN1	-0.809	0.005
RVSP	LGR5	-0.772	0.009

EV: Extracellular vesicle; LVEDV: left ventricular end-diastolic volume; LVEF: left ventricular ejection fraction; LVESV: left ventricular end-systolic volume; QRS: QRS duration; RV: right ventricular; RVEDV: right ventricular end-diastolic volume; RVEF: right ventricular ejection fraction; RVSP: right ventricular systolic pressure.

### EV subpopulation alteration

We performed cluster analysis on EVs based on proteomic characteristics to identify EV subpopulations. We calculated the proportion of each subpopulation and compared the protein expression levels across them. In total, we classified the EVs into 14 subpopulations and visualized the characteristic proteins of each subpopulation using a heatmap [Figure 4A]. Subsequently, two dimensionality reduction and visualization algorithms, t-SNE and UMAP, were employed to visualize the single-EV protein expression data. The t-SNE and UMAP scatter plots revealed that EVs across all samples primarily clustered in Cluster 1 (18.79%), Cluster 2 (27.79%), Cluster 4 (20.5%), and Cluster 10 (10.23%) [Supplementary Figure 8A and B]. Individual sample t-SNE and UMAP visualizations further showed the distribution of EV subpopulations across the *N*, T1, T2, and T3 groups [Supplementary Figure 8C and D]. Subsequently, we performed clustering analysis on EVs from the *N*, T1, T2, and T3 groups [Figure 4B-E].

**Figure 4:**
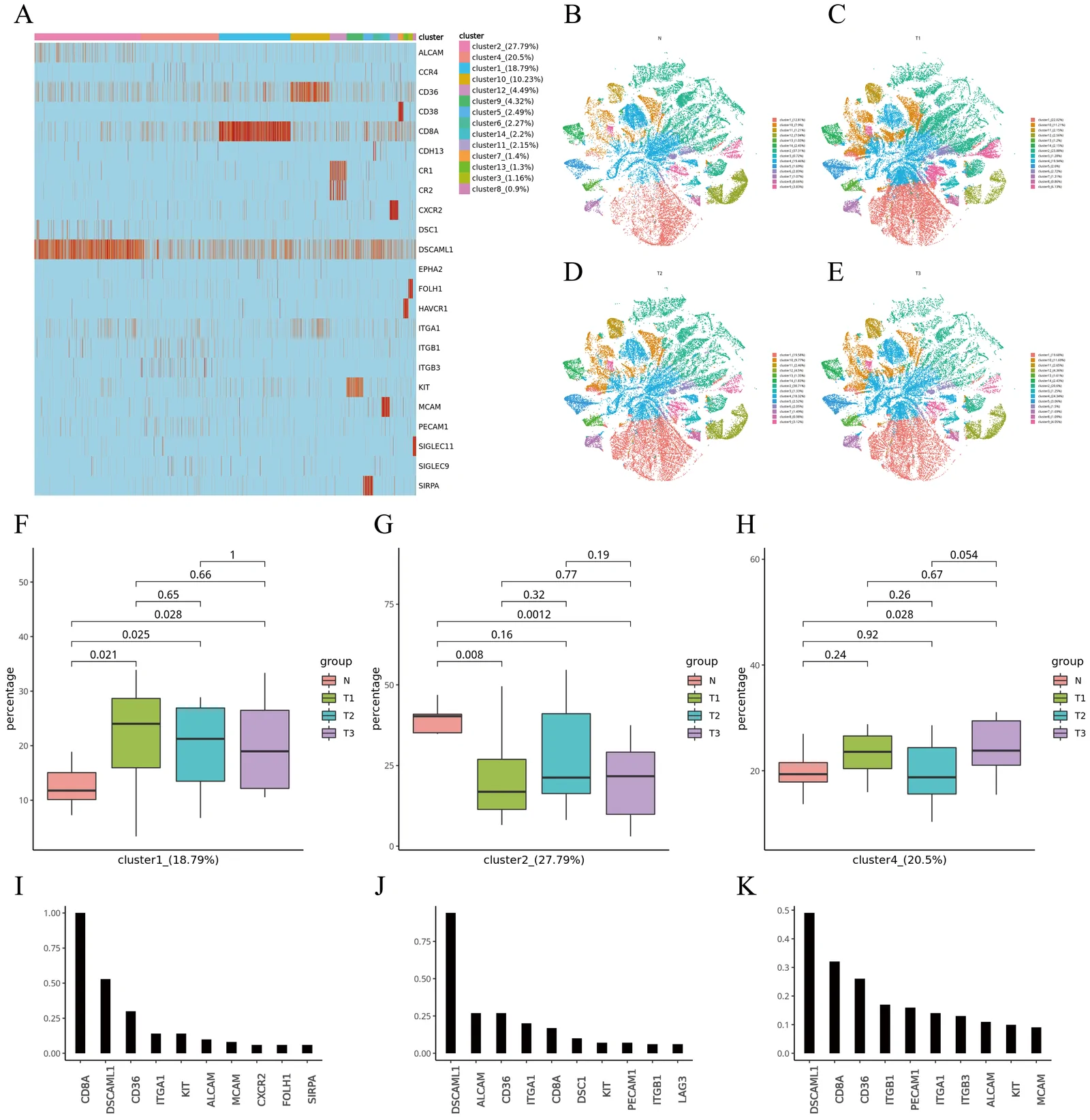
Identification and characterization of EV subpopulations. The heatmap visualizes the protein expression profiles across EV subpopulations (A). Each column represents a distinct EV, while each row corresponds to an individual protein. Different colors denote separate EV subpopulations. TSNE scatter plots of EV subpopulations across groups *N*, T1, T2, and T3 (B-E). Different colors represent distinct subpopulations, with percentages in parentheses indicating the proportion of each subpopulation. Boxplots of Cluster 1, Cluster 2, and Cluster 4 subpopulations across different samples (F-H). The x-axis represents different groups, while the y-axis indicates the proportion of a specific subpopulation across these groups. The top 10 proteins in Cluster 1, Cluster 2, and Cluster 4. The x-axis denotes protein names, while the y-axis represents the proportion of each protein within the subpopulation (I-K). EV: Extracellular vesicle; TSNE: T-distributed stochastic neighbor embedding.

We then performed differential expression analysis of each EV subpopulation’s proportion and protein expression levels across groups. Cluster 1 shows a significantly lower proportion in the *N* group (12.81%) compared to T1 (22.02%), T2 (19.58%), and T3 (19.68%) (*P* < 0.05) [Figure 4F]. Conversely, Cluster 2 has a significantly higher proportion in the *N* group (37.31%) compared to T1 (23.88%) and T3 (20.6%) (*P* < 0.05) [Figure 4G]. Additionally, the proportion of Cluster 4 is significantly higher in the T3 group (24.34%) compared to the *N* group (19.46%) (*P* < 0.05) [Figure 4H]. Subsequently, we performed a detailed analysis of each subpopulation and listed the top ten most abundantly expressed proteins. Notably, the top three proteins in Cluster 1, Cluster 2, and Cluster 4 were CD8A, DSCAML1, and CD36; DSCAML1, ALCAM, and CD36; and DSCAML1, CD8A, and CD36, respectively [Figure 4I-K]. Moreover, Clusters 3 and 5 were significantly less abundant in the control group compared to the disease group (*P* < 0.05) [Supplementary Figure 9A and B]. In contrast, Clusters 7, 8, and 11 were markedly more abundant in the T2 and T3 groups than in the control group (*P* < 0.05) [Supplementary Figure 9C-E]. Conversely, Cluster 12 was significantly more abundant in the control group than in the disease group (*P* < 0.05) [Supplementary Figure 9F]. The top ten proteins for Clusters 3, 5, 7, 8, 11, and 12 are shown in Supplementary Figure 9G-L.

## DISCUSSION

This study is the first to utilize PBA technology for dynamic monitoring of perioperative plasma EVs in TPVR patients, revealing the surface protein profiles and subpopulation characteristics of plasma EVs. The study identified significant DEPs between the *N* and T1 groups, as well as between the T1 and T2/T3 groups. Simultaneously, EV subpopulation analysis revealed dynamic changes in EV subpopulations at different time points, providing new clues for understanding the mechanisms of RV remodeling after TPVR.

We found that proteins such as LAG3 were downregulated in the preoperative group but rapidly rebounded within 1 day postoperatively. LAG3, a new target in cancer immunotherapy, is expressed across multiple leukocyte subsets, including T cells^[19]^. Its dynamic changes may be associated with alterations in T cell immune status before and after TPVR, although direct evidence from tissue or functional studies is not yet available. Mulholland *et al*. discovered that while LAG3 deficiency or blockade does not affect the size of atherosclerotic plaques, the absence of LAG3 leads to a twofold increase in T cell density within the plaques^[20]^. CD8A is a critical marker of cytotoxic T cells. Its elevated expression directly indicates an enhanced cellular immune response. Research has shown that CD8^+^ T cells can exacerbate adverse remodeling after myocardial ischemia by releasing granzyme B and other pathways^[21]^. Bartoli-Leonard *et al*. collected RV tissue from pediatric CHD patients undergoing cardiac surgery and performed transcriptomic and functional network analyses^[22]^. The results demonstrated a significant increase in the cytotoxic T cell marker CD8A (*P* = 0.0006) in CHD patients, accompanied by positive regulation between the immune system and cytokine signaling clusters. Our findings are consistent with the possibility that chronic pressure and volume overload in PR may be associated with immune processes involving cytotoxic T cells, but causal evidence requires further experimental validation. Postoperatively, as hemodynamic abnormalities are corrected, changes in the expression of these immune-related proteins may reflect the attenuation or transformation of the inflammatory response.

Regarding myocardial injury and repair, ALCAM, an important cell adhesion molecule, has been identified as an independent predictor of cardiovascular mortality in patients with acute coronary syndrome [HR 1.45 (1.16-1.82), *P* = 0.0012]^[23]^. Delgado *et al*. found that ALCAM is highly enriched in cardiac Purkinje cells, suggesting that the development of the ventricular conduction system relies on neuronal cell adhesion molecules^[24]^. The reduced preoperative expression observed in PR patients likely indicates dysfunction in intercellular signaling among RV endothelial cells and cardiomyocytes, resulting from prolonged volume overload. Conversely, the prompt postoperative upregulation suggests a concurrent activation of endothelial repair mechanisms and the recovery of cellular crosstalk. Jha *et al*. discovered that downregulation of LGR5 expression can inhibit cardiomyocyte differentiation while promoting endothelial cell differentiation in human pluripotent stem cells^[25]^. Huang *et al*. found that disruption of the immune checkpoints PD-1 and CTLA-4 leads to increased T lymphocyte infiltration and elevated pro-inflammatory cytokine levels^[26]^, which accelerate atherosclerotic plaque formation and promote the progression of HF^[21]^.

While traditional biomarker research often focuses on single molecules, the function of EVs is typically dictated by the combination of molecules they carry. This study is the first to depict the complex co-expression network of membrane proteins at the single-EV level in TPVR patients during the perioperative period using PBA technology. We found that, compared with the control group, the disease group exhibited a significant downregulation of the protein combination of ALCAM&CD36&DSCAML1&PECAM1 (*P* < 0.001; AUC > 0.94), while the expression levels of CD36&CD8A&DSCAML1&TACSTD2 was significantly upwnregulated (*P* < 0.05). These protein sets are primarily involved in cellular adhesion, stress response, and immune system processes, and their coordinated changes may more precisely reflect the pathological state of the RV microenvironment than individual proteins. Functional enrichment analysis revealed that the protein combinations associated with RV remodeling were significantly enriched in the PI3K-Akt signaling pathway, which is a central pathway promoting angiogenesis and regulating myocardial remodeling^[27]^. The abnormal activation of this pathway before surgery reflects the pathological hypertrophy of the RV in response to volume overload. Postoperatively, alterations in the abundance of associated proteins suggest that this pathway may be transitioning from pathological remodeling towards physiological reversal.

Flow cytometry was used to validate the differential expression of five candidate proteins, ALCAM, LGR5, LAG3, NECTIN1, and CD8A, in plasma EV samples from the *N*, T1, and T2 groups. As shown in Supplementary Figures 6 and 7, none of these proteins showed statistically significant differences among the three groups. Several factors may explain why the flow cytometry results did not replicate the PBA findings. First, the two technologies differ substantially in their analytical principles. PBA captures individual EVs on a surface and enables high-plex profiling of multiple surface proteins on single vesicles, while conventional flow cytometry is typically limited by its detection threshold and may not resolve smaller EVs or low-abundance signals^[28]^. Recent studies have shown that quantitative discrepancies across different analytical platforms can reach up to two orders of magnitude^[29]^. Second, the sample size for flow cytometry was even smaller (*N* = 7, T1 = 5, T2 = 7) due to limited residual sample volumes, which substantially reduces statistical power. Third, the panel of five antibodies used for validation represents only a fraction of the 260-protein PBA panel, and may not fully capture the combinatorial protein signatures that distinguish different disease states.

The study identified 14 distinct functional EV subpopulations and revealed their dynamic evolution throughout the perioperative period. Cluster 1, characterized by CD8A expression, was significantly enriched in the patient cohort, whereas Cluster 2, dominated by DSCAML1, was predominant in the control group. Studies have demonstrated that source-tracing analysis of plasma EVs, combined with machine learning, can resolve the genetic signatures of cardiac-derived EVs^[30]^. This study demonstrates that ALCAM primarily originates from cardiac progenitor cells and cardiac cells^[31]^. These findings highlight the tremendous advantages of PBA technology in dissecting disease heterogeneity and identifying composite biomarkers. This suggests that in the future, the primary cellular origins of proteins could be more precisely identified through membrane protein characteristics.

This study has several limitations. First, the sample size is relatively small, with only ten TPVR patients and ten healthy controls. This limits statistical power and increases the risk of overfitting and false positive discoveries. Second, due to the relatively low incidence of severe PR requiring TPVR and strict quality control, we were unable to split the data into training and test sets or enroll an external validation cohort. Third, we lacked tissue samples, such as myocardial biopsies to confirm protein expression at the tissue level, and no *in vivo* or *in vitro* functional experiments were conducted to establish causal relationships. Finally, this study lacks long-term follow-up data, making it impossible to assess the relationship between DEPs and patients’ long-term prognosis. It should also be noted that the NTA, TEM, and WB characterization data were obtained from control group plasma samples processed separately from the PBA experiment, with no biological replicates included, as these assays were performed for EV identification. Future research directions should include: (1) Expanding the sample size and conducting multicenter validation to establish a reliable diagnostic model; (2) Investigating the impact on cardiomyocytes, fibroblasts, and endothelial cells in both *in vitro* and *in vivo* models to elucidate the specific mechanisms involved in RV remodeling; (3) Extending the follow-up period to investigate the association between perioperative EV characteristics and RV functional recovery, as well as major adverse cardiovascular events, at 1 year postoperative and beyond.

In conclusion, this study is the first to apply single-EV membrane proteomics technology to map the dynamic plasma exosome protein landscape and subpopulation trajectories throughout the perioperative period of TPVR, providing candidate EV features associated with RV remodeling. Compared with the control group, preoperative patient plasma EVs exhibited significantly downregulated expression of DSCAML1, ALCAM, and LAG3, whereas CD8A, NECTIN1, and LGR5 were significantly upregulated, demonstrating exploratory discrimination in this pilot cohort. Subsequent flow cytometry validation did not yield statistically significant results. Multi-protein combinatorial analysis revealed that the expression levels of the ALCAM&CD36&DSCAML1&PECAM1 protein complex were significantly upregulated in the control group, while the CD36&CD8A&DSCAML1&TACSTD2 complex was markedly downregulated. GO analysis revealed significant enrichment in pathways related to response to stress, side of membrane, and protein binding. KEGG pathway analysis indicated enrichment in the PI3K-Akt signaling pathway. Furthermore, several EV proteins showed significant correlations with preoperative and postoperative clinical parameters, including indices of both RV and LV remodeling. EV subpopulation analysis revealed that EVs in the disease group were dominated by CD8A protein expression, whereas those in the control group were predominantly characterized by DSCAML1 protein expression.

## DECLARATIONS

### Availability of data and materials

The raw data of single-EV surface proteins generated in this study have been deposited in the Figshare repository (https://doi.org/10.6084/m9.figshare.32588316).

### Copyright

© The Author(s) 2026.
