# Supplementary material for: Dynamic profiles and subpopulation characteristics of extracellular vesicles in patients undergoing transcatheter pulmonary valve replacement
Source: Extracell Vesicles Circ Nucl Acids. 2026 Aug 18;7(3):1297–313. doi: 10.20517/evcna.2026.24 (PMC13678174; doi:10.20517/evcna.2026.24)
Supplement: Supplementary file 1 [file evcna-7-3-1297-SupplementaryMaterials.pdf]

Supplementary Materials

Dynamic profiles and subpopulation characteristics of extracellular vesicles in patients undergoing transcatheter pulmonary valve replacement

Runzhang Liang[1,2,#], Jiaxiong Wu[1,2,#], Naijimuding Abudurexiti[3,#], Jing Ling[1,2,4], Zirui Peng[2,4], Canxin Wang[1,2], Jinxin Li[1,2], Yong Zhang[1,2,4], Haiyun Yuan[1,2,4], Shusheng Wen[1,2,4]

[1]Department of Cardiovascular Surgery, Guangdong Provincial People's Hospital (Guangdong Academy of Medical Sciences), Southern Medical University, Guangzhou 510080, Guangdong, China.

[2]Guangdong Provincial Key Laboratory of South China Structural Heart Disease, Guangzhou 510080, Guangdong, China.

[3]Department of Cardiovascular Surgery, Guangdong Provincial Hospital of Chinese Medicine, The Second Affiliated Hospital of Guangzhou University of Chinese Medicine, Guangzhou 510120, Guangdong, China.

[4]Department of Cardiovascular Surgery, Guangdong Cardiovascular Institute, Guangdong Provincial People's Hospital, Guangdong Academy of Medical Sciences, Guangzhou 510080, Guangdong, China.

[#]These authors contributed equally to this work.

Correspondence to: Prof. Shusheng Wen, Department of Cardiovascular Surgery, Guangdong Provincial People's Hospital (Guangdong Academy of Medical Sciences), Southern Medical University, Guangzhou 510080, Guangdong, China. E-mail: wenshusheng@gdph.org.cn

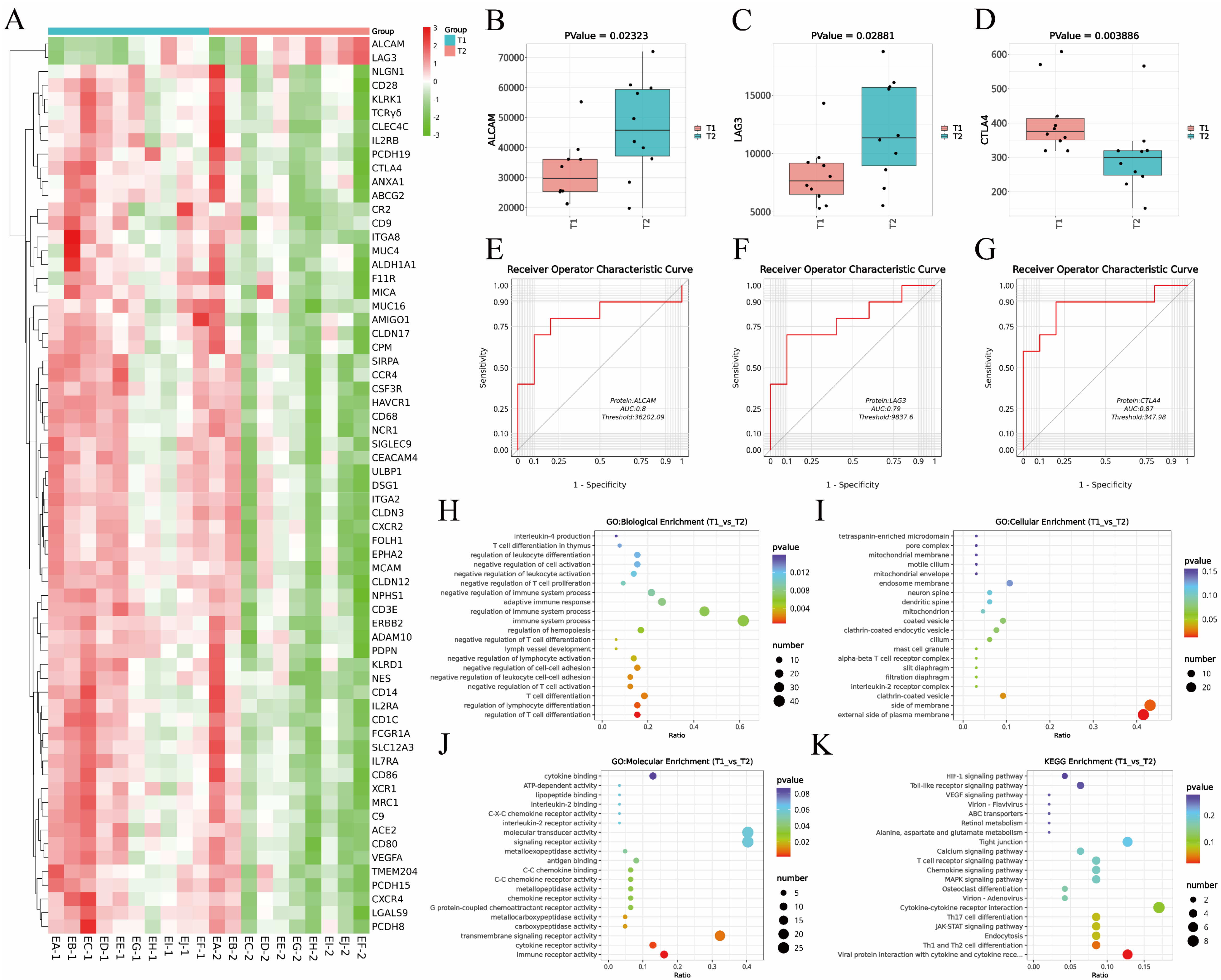

Supplementary Figure 1. Differential expression analysis of individual proteins between the T1 group and the T2 group. The heatmap displays the DEPs in EVs between the T1 and T2 groups (A). Histograms of DEPs (B-D). ROC curve analyses of DEPs (E-G). GO pathway enrichment analysis of DEPs (H-J). KEGG pathway enrichment analysis of DEPs (K). DEPs: Differentially expressed proteins; EV: Extracellular vesicle; GO: Gene Ontology; KEGG: Kyoto Encyclopedia of Genes and Genomes; ROC: Receiver operating characteristic.

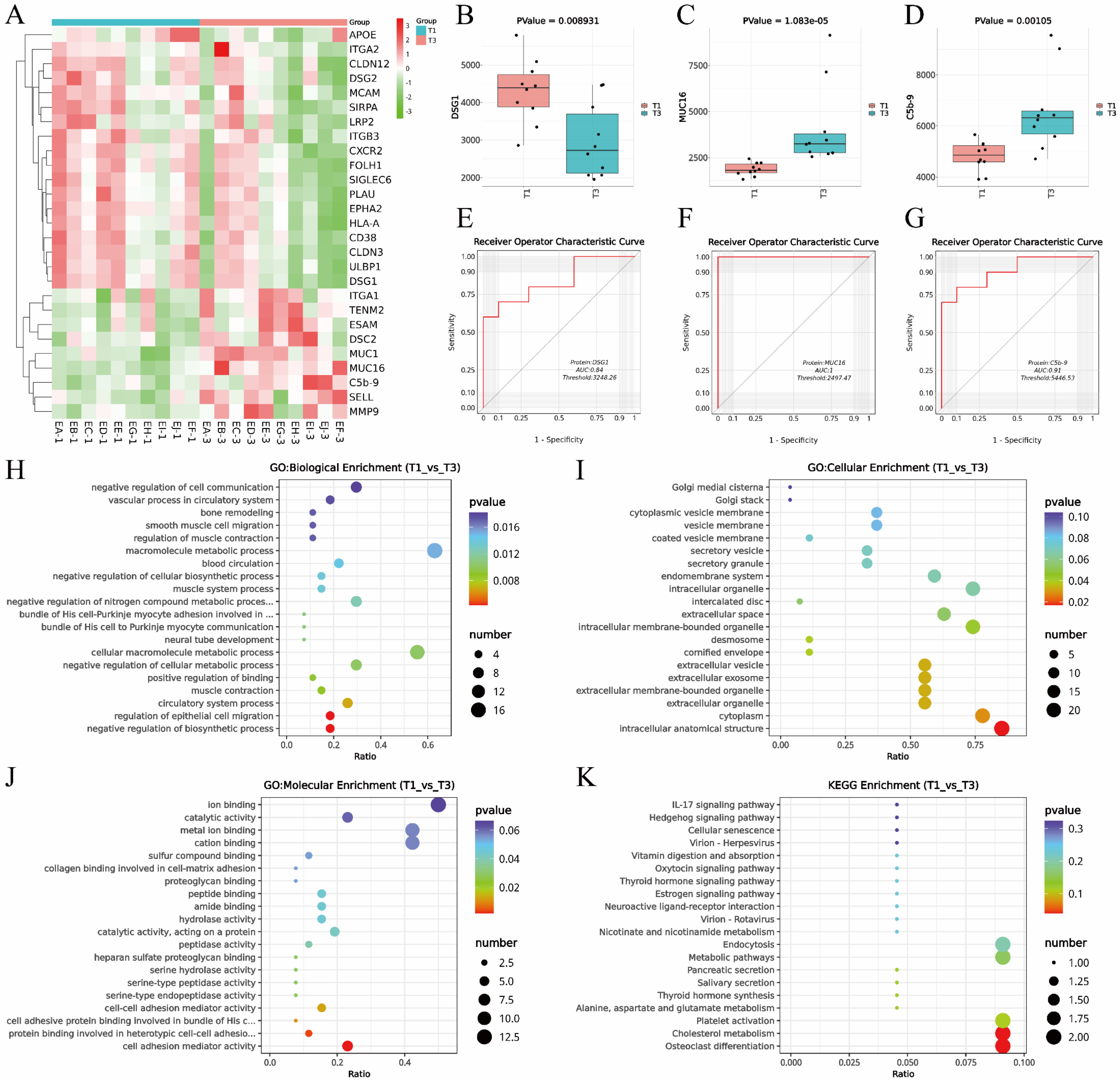

Supplementary Figure 2. Differential expression analysis of individual proteins between the T1 group and the T3 group. The heatmap displays the DEPs in EVs between the T1 and T3 groups (A). Histograms of DEPs (B-D). ROC curve analyses of DEPs (E-G). GO pathway enrichment analysis of DEPs (H-J). KEGG pathway enrichment analysis of DEPs (K). DEPs: Differentially expressed proteins; EV: Extracellular vesicle; GO: Gene Ontology; KEGG: Kyoto Encyclopedia of Genes and Genomes; ROC: Receiver operating characteristic.

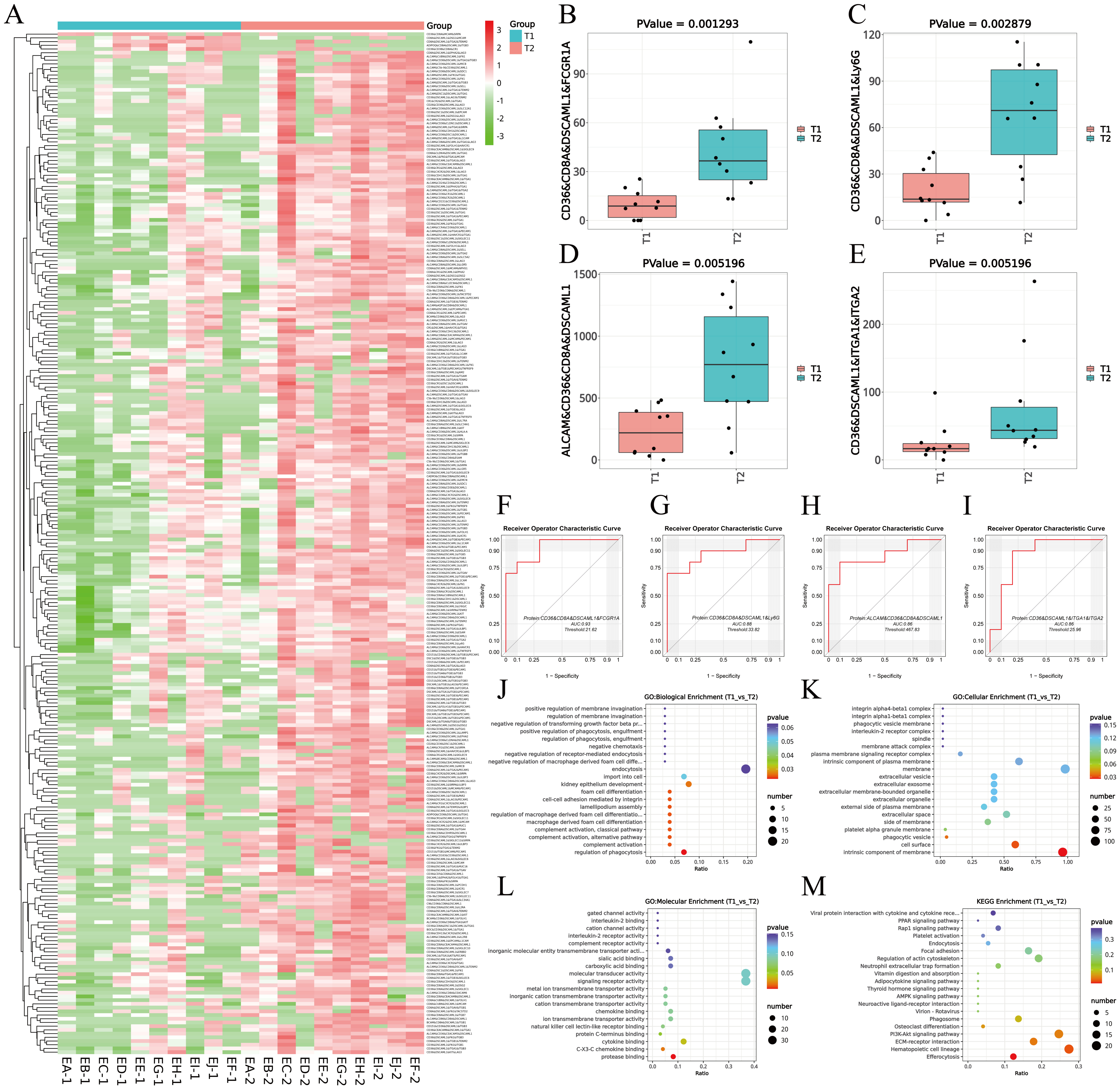

Supplementary Figure 3. Differential expression analysis of protein combinations between the T1 group and the T2 group. The heatmap displays the differentially expressed protein combinations in EVs between the T1 and T2 groups (A). Histograms of differentially expressed protein combinations (B-E). ROC curve analyses of differentially expressed protein combinations (F-I). GO pathway enrichment analysis of differentially expressed protein combinations (J-L). KEGG pathway enrichment analysis of differentially expressed protein combinations (M). EV: Extracellular vesicle; GO: Gene Ontology; KEGG: Kyoto Encyclopedia of Genes and Genomes; ROC: Receiver operating characteristic.

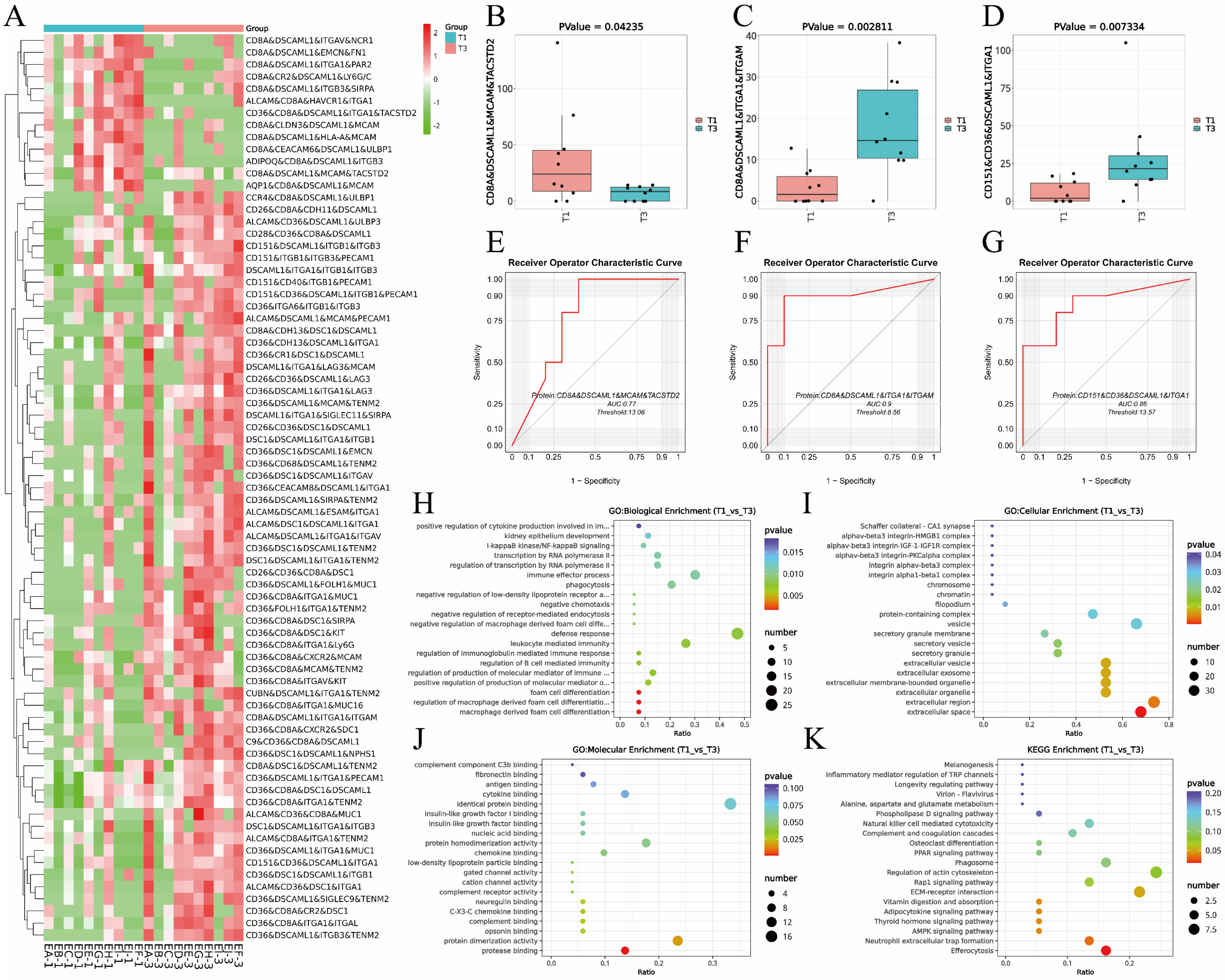

Supplementary Figure 4. Differential expression analysis of protein combinations between the T1 group and the T3 group. The heatmap displays the differentially expressed protein combinations in EVs between the T1 and T3 groups (A). Histograms of differentially expressed protein combinations (B-D). ROC curve analyses of differentially expressed protein combinations (E-G). GO pathway enrichment analysis of differentially expressed protein combinations (H-J). KEGG pathway enrichment analysis of differentially expressed protein combinations (K). EV: Extracellular vesicle; GO: Gene Ontology; KEGG: Kyoto Encyclopedia of Genes and Genomes; ROC: Receiver operating characteristic.

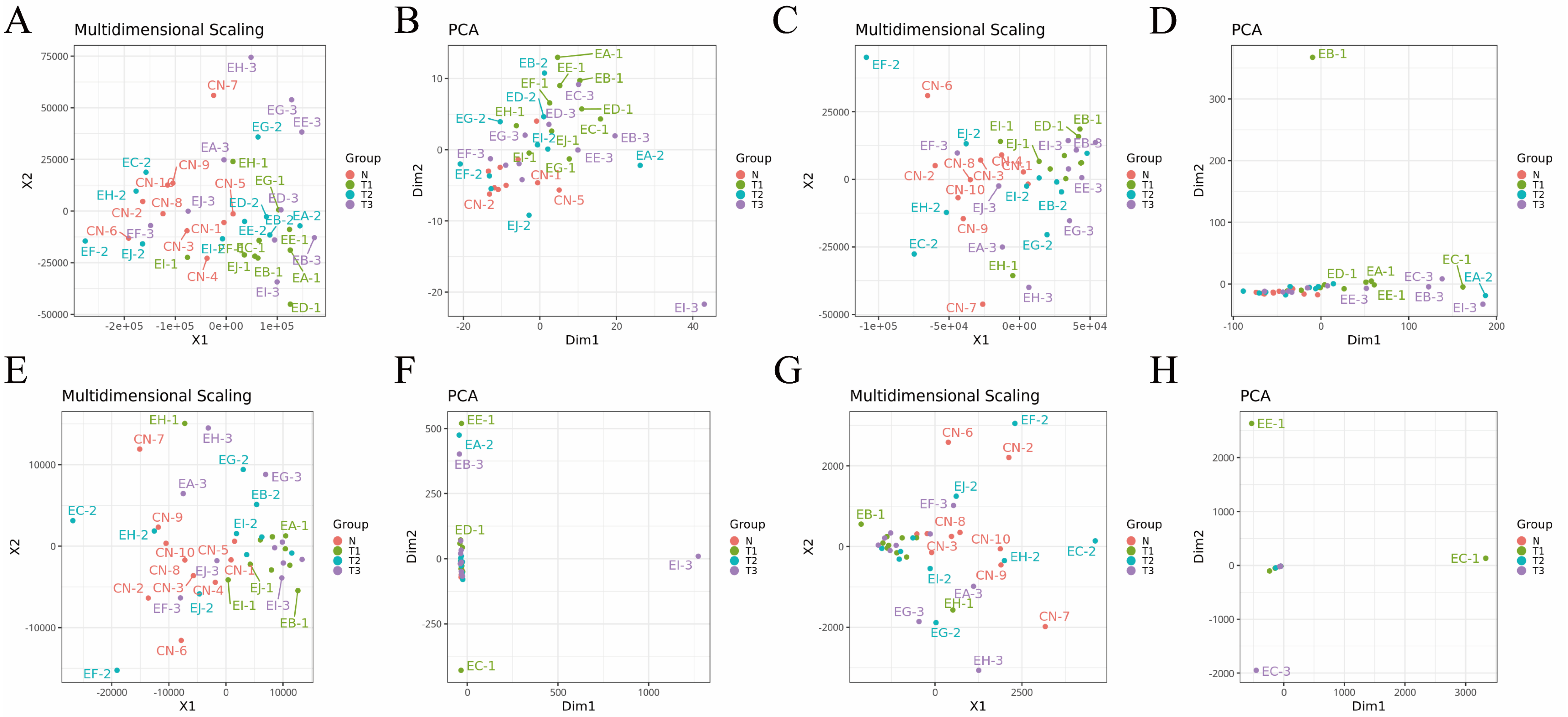

Supplementary Figure 5. PCA and MDS were performed on co-expression patterns formed by single protein (A and B), two proteins (C and D), three proteins (E and F), and four or more proteins (G and H) at the single-EV level. Each dot represents a sample, with colors indicating different groups, N group (Red), T1 group (Green), T2 group (Blue), and T3 group (Purple). EV: Extracellular vesicle; MDS: Multidimensional scaling; PCA: Principal component analysis.

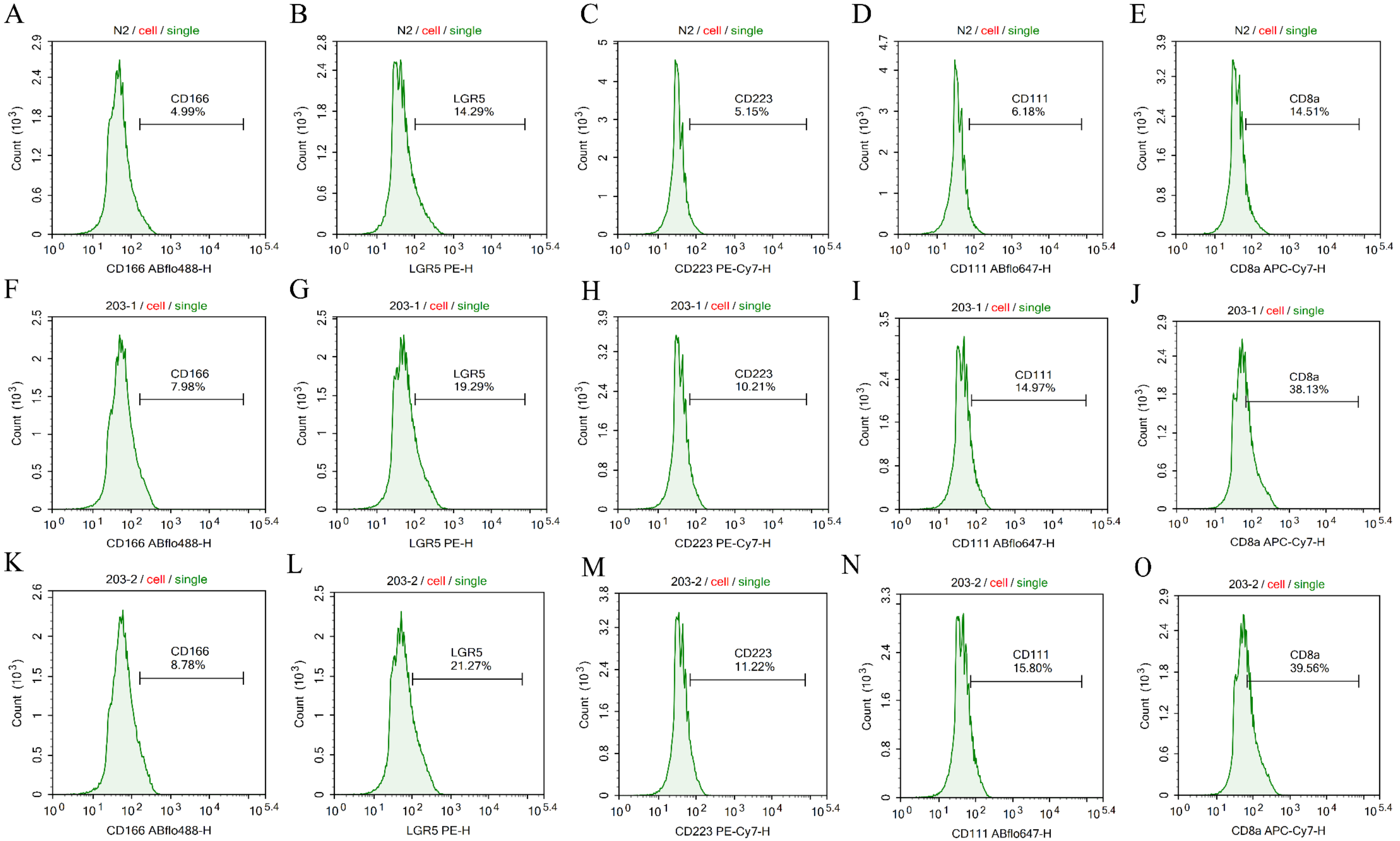

Supplementary Figure 6. Flow cytometric analysis of EVs surface protein expression in representative samples from N (A-E), T1 (F-J), and T2 (K-O) groups. Each histogram shows the percentage of EVs positive for CD166, LGR5, CD223, CD111, and CD8A. The x-axis represents fluorescence intensity and the y-axis shows normalized event counts. EV: Extracellular vesicle.

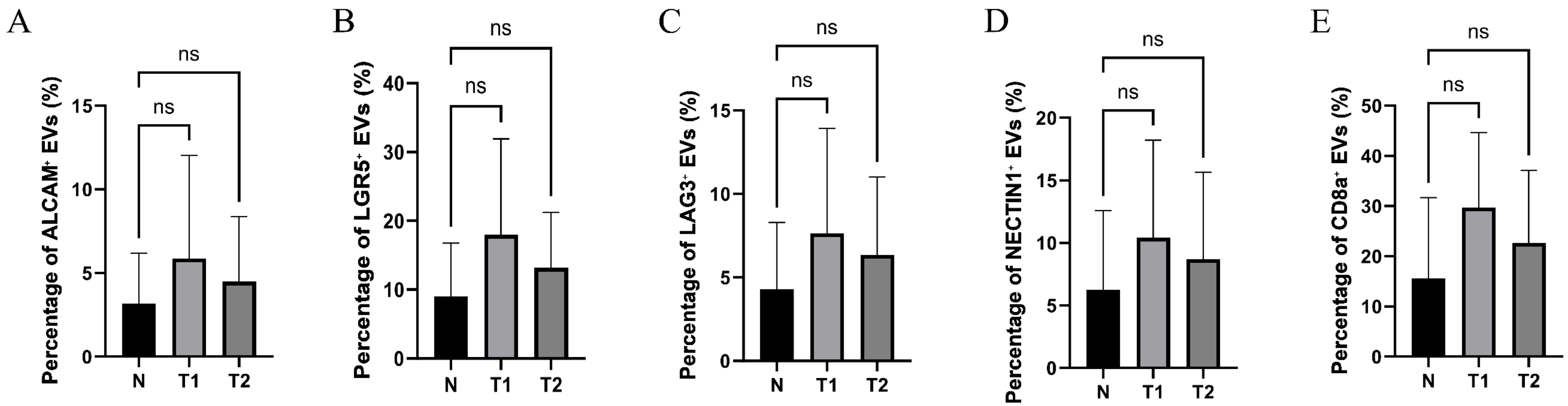

sss

Supplementary Figure 7. Flow cytometric quantification of EV surface protein expression in the N, T1, and T2 groups. The percentages of EVs positive for (A) ALCAM, (B) LGR5, (C) LAG3, (D) NECTIN1, and (E) CD8a were compared among the three groups. Statistical significance was determined by one-way ANOVA followed by a post-hoc test with appropriate correction for multiple comparisons. $^*P < 0.05$, $^{**}P < 0.01$, $^{***}P < 0.001$. ns indicates not significant. EV: Extracellular vesicle.

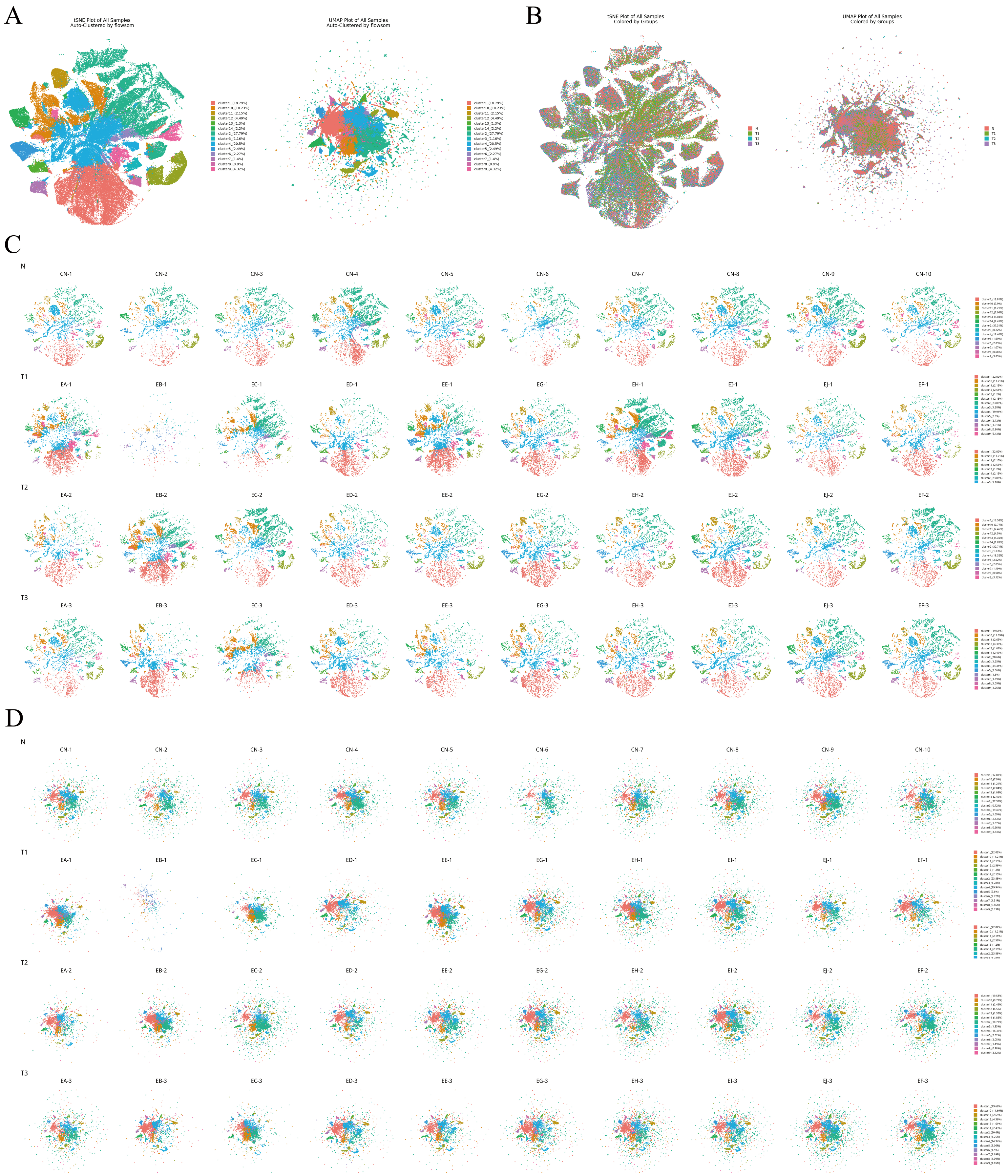

Supplementary Figure 8. Identification and characterization of EV subpopulations. Identification of EV subpopulations across all samples (A). T-SNE scatter plot (left) and UMAP scatter plot (right) of different subpopulations. Identification of EV subpopulations across all groups (B). T-SNE scatter plot (left) and UMAP scatter plot (right) of different groups. T-SNE scatter plot of different samples (C). UMAP scatter plot of different samples (D). Each dot represents a single EV. Different colors indicate distinct EV subgroups, and the value in parentheses denotes the proportion of that subpopulation relative to the total across all subgroups. EV: Extracellular vesicle; TSNE: T-distributed stochastic neighbor embedding. UMAP: Uniform Manifold Approximation and Projection.

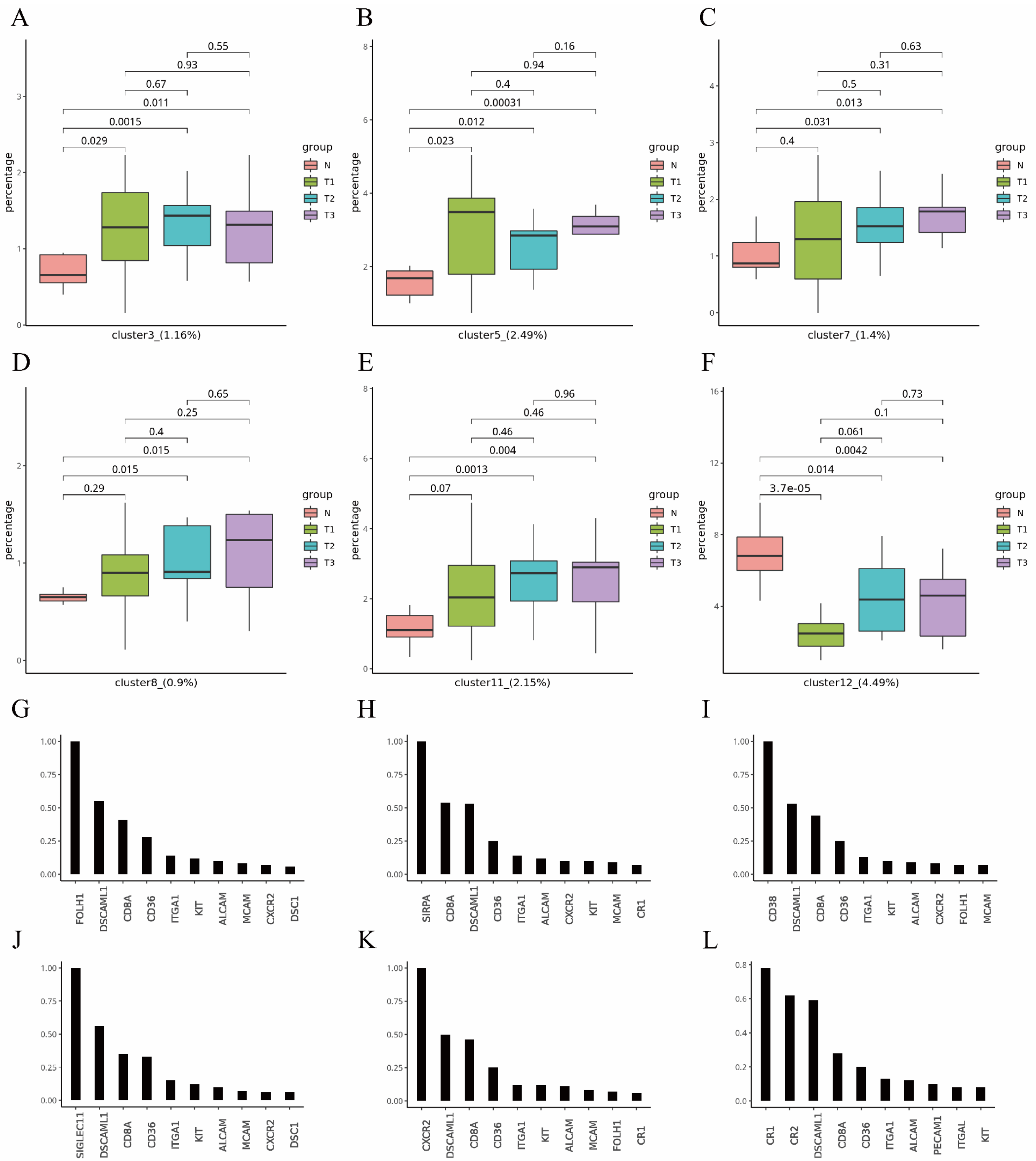

Supplementary Figure 9. Box plots of the Cluster 3, Cluster 5, Cluster 7, Cluster 8, Cluster 11, and Cluster 12 subpopulations across different groups (A-F). The x-axis represents different groups, while the y-axis indicates the proportion of a specific subpopulation across these groups. Top 10 proteins of the subpopulations Cluster 3, Cluster 5, Cluster 7, Cluster 8, Cluster 11, and Cluster 12 (G-L). The x-axis denotes protein names, while the y-axis represents the proportion of each protein within the subpopulation.
